# Effective Dressing: Development of N-Carboxyethyl Chitosan/Oxidized Locust Bean Gum Scaffolds

**DOI:** 10.1021/acsomega.5c00521

**Published:** 2025-04-21

**Authors:** Luis F. S. Araujo, Carlos R. do N. Ferreira, Gisele S. de Araújo, Ana J. Araújo, José D. B. Marinho-Filho, Ana B. N. Lima, André T. de F. F. Dias, Matheus da S. Campelo, Renata F. de C. Leitão, Maria E. N. P. Ribeiro, Regina C. M. de Paula, Judith P. A. Feitosa, Jeanny da S. Maciel

**Affiliations:** † Departamento de Química Orgânica e Inorgânica, Universidade Federal do Ceará, Av. Mister Hull s/n, 60455-760 Fortaleza, Brazil; ‡ Laboratório de Cultura de Células do Delta, Universidade Federal do Delta do Parnaíba, Av. São Sebastião 2819, 64202-020 Parnaíba, Brazil; § Departamento de Morfologia, Faculdade de Medicina, Universidade Federal do Ceará, R. Delmiro de Farias s/n, 60430-170 Fortaleza, Brazil

## Abstract

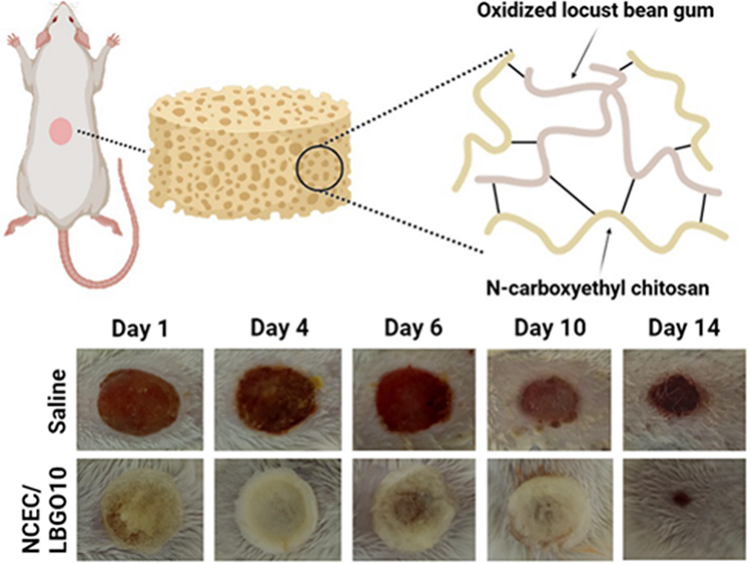

This study aimed to prepare, characterize, and evaluate the wound healing activity of scaffolds based on N-carboxyethyl chitosan (NCEC) and oxidized locust bean gum (LBGO) synthesized through the Schiff base reaction. NCEC was prepared by Michael addition reaction, and LBGO (degrees of oxidation: 10, 30, and 50%) was synthesized using sodium periodate. These reactions were confirmed by using spectroscopic and titrimetric techniques. The scaffolds were prepared by freeze-drying and characterized regarding morphology, porosity, mechanical properties, cytotoxicity, and wound healing activity. The spectroscopic and titrimetric data demonstrated that the chemical modifications of chitosan and locust bean gum were successfully carried out, as well as the formation of the Schiff base to obtain the scaffolds. The biomaterials presented a porous appearance, with empty spaces ranging from 37 to 76%, whose pores had diameters between 73 and 268 μm. Swelling capacity increased with the increase in the degree of oxidation of LBGO (2300−3600%), indicating that this parameter is capable of modulating the morphology and mechanical properties of the biomaterial, which was able to maintain its structure for 28 days in the in vitro degradation study. Cytotoxicity assays using L929 fibroblast cells demonstrated that all scaffolds were nontoxic (cell viability between 98 and 82%), confirming their biocompatibility. In vivo assay showed that scaffolds accelerate the healing process, increase the thickness of the epidermis and dermis, and attenuate tissue oxidative stress by reducing the levels of pro-oxidant mediators. This study demonstrates that NCEC/LBGO scaffolds are effective biomaterials for wound dressing applications.

## 1 Introduction

Biomaterials, materials designed to interact with biological systems, play a crucial role in the fields of medicine and tissue engineering as they have the potential to enhance both the quality and longevity of human life. These biomaterials are derived from diverse materials including metals, ceramics, and synthetic and natural polymers. The application of biomaterials is quite broad in the medical disciplines, such as cardiovascular medicine, orthopedics, dentistry, plastic surgery, ophthalmology, wound healing, and tissue engineering.^1^ Tissue engineering is dedicated to repairing and restoring the structural functionality of impaired tissues.^2^ Concisely, the wound healing process is typically accomplished through the implementation of one of three strategies: (a) employing cells or cell substitutes as replacements for damaged tissue; (b) utilizing acellular biomaterials that can induce tissue regeneration; (c) combining both approaches with a temporary porous scaffold that facilitates cell reorganization and the production of their extracellular matrix.^3^ Scaffolds are biomaterials consisting of three-dimensional networks formed by cross-linking hydrophilic polymers through techniques involving covalent bonds, physical interactions, or a combination of both. Commonly employed cross-linking methods include Michael additions, click reactions, and Schiff base formation reactions.^4^

For scaffold preparation, the use of natural polymers has been used extensively due to their biocompatibility, biodegradability, and low immunogenic properties.^2^ One of the most used natural polymers is chitosan, a partially deacetylated derivative of chitin, which is composed of N-acetyl-d-glucosamine and 2-amino-2-deoxy-d-glycopyranose units linked through β-(1→4) bonds. This polysaccharide possesses nontoxic, biocompatible, and biodegradable properties, gradually degrading into amino sugars that are fully absorbed by the body.^5^ Consequently, chitosan is indeed considered a suitable biomaterial for tissue engineering. However, this polysaccharide has a major drawback regarding its solubility, which is limited to acidic media (pH < 6). This solubility obstacle is quite challenging since the physiological pH of the body is 7.4, making chitosan insoluble and necessitating structural modifications. Several reactions have been reported in the literature for producing chitosan derivatives with biomedical applications, including amino group quaternization, carboxymethylation, and the addition of phosphate and sulfate groups.^6^ One of the most studied derivatives is water-soluble N-carboxyethyl chitosan (NCEC). Recent studies showed that the synthesis of NCEC can be achieved by performing a Michael addition reaction of chitosan with acrylic acid in an aqueous medium.^7^

Other natural polymers used for scaffold synthesis are galactomannans, polysaccharides composed of a main chain of β-D-mannopyranosidic units linked by (1 → 4) bonds, with α-D-galactopyranosidic side groups attached through (1 → 6) linkages.^8^ Locust bean gum (LBG) is a galactomannan extracted from carob tree (*Ceratonia siliqua*) seeds. Due to its nontoxic nature, it finds extensive use in the food and pharmaceutical industries. Its thickening and gelling properties are employed in the production of various formulations.^9^ Galactomannans can be modified to enhance their application as biomaterials. Oxidation,^10^ carboxymethylation,^11^ sulfation, and carboxylation,^12^ are examples of modifications carried out on galactomannans. The vicinal diols present in the structure of LBG enable oxidation reactions using periodate ions, which attack the vicinal diols, resulting in the cleavage of C−C bonds and the formation of aldehyde groups.^10^ The oxidized locust bean gum (LBGO) can form hydrogels by cross-linking its aldehyde groups with the amino groups present in NCEC via a Schiff base formation reaction.

The Schiff base reaction involves the nucleophilic attack of the nitrogen atom in the amino group on the aldehyde’s electrophilic carbon, forming an imine bond. This reaction occurs in an aqueous environment under physiological conditions, resulting in the production of nontoxic products. As a result, these materials are considered safer for the production of biomaterials.^13^ Hydrogels formed through Schiff base reactions are widely employed in diverse biomedical applications, such as drug release, tissue regeneration, wound healing, tissue adhesives, bioprinting, and biosensors.^14^ Based on the above, this study proposes the preparation and characterization of scaffolds by cross-linking N-carboxyethyl chitosan with oxidized locust bean gum through the Schiff base reaction. In vitro cytotoxicity test and in vivo wound healing experiments were conducted to assess their potential as biomaterials.

## 2 Materials and Methods

### 2.1 Materials

Chitosan was supplied by Polymar, with an 81% degree of deacetylation. Locust bean gum from the seeds of*Ceratonia siliqua* was obtained from Sigma-Aldrich. Ethyl alcohol, acetone, and acrylic acid were obtained by Synth. Ethylene glycol, hydroxylamine hydrochloride, sodium hydroxide, dibasic sodium phosphate, and monobasic potassium phosphate were obtained by Vetec and sodium periodate by Dinâmica. 5,5-Dithio-bis (2-nitrobenzoic acid) (DTNB) and N-1-naphthylenediamine (NEED) were purchased from Sigma-Aldrich.

### 2.2 Synthesis of N-Carboxyethyl Chitosan (NCEC)

The water-soluble chitosan derivative was synthesized following the methodology described in Ferreira et al.,^15^ with the goal of achieving a theoretical degree of substitution of 12.5% of monosaccharide units. In a round-bottom flask, 50 mL of distilled water, 0.365 mL of acrylic acid, and 1.0 g of purified chitosan were added. The mixture was continuously stirred for 72 h at 50 °C in a glycerin bath. After that, the pH was adjusted to 10−12 using a 1.0 mol L^−1^ sodium hydroxide (NaOH) solution. The mixture was then precipitated in ethanol with a volume ratio of 2:1 (ethanol:mixture) and washed with ethanol and acetone, and the solid was dried using hot air blowing.

### 2.3 Locust Bean Gum Oxidation

The partial oxidation of locust bean gum (LBG) was performed according to the methodology described in da Silva et al.,^16^ with some modifications. Purified LBG (3.0 g) was added to 600 mL of distilled water at a concentration of 0.5% (w v^−1^) and stirred for 1 h at room temperature. The mixture was then heated to 80 °C for 30 min, followed by 24 h of stirring at room temperature. To achieve theoretical degrees of oxidation of 10, 30, and 50% of the monosaccharide units (162 g mol^−1^), 3.7, 11.1, and 18.5 mL of 0.5 mol L^−1^ sodium periodate (NaIO_4_) were added to the respective solution. The derivatives were designated as LBGO10, LBGO30, and LBGO50. The system was kept under agitation for 24 h and covered with aluminum foil to prevent photoinduced decomposition of the periodate ion, as described by Dawlee et al.^17^ To halt the reaction, 1 mL of ethylene glycol (CH_2_OH)_2_ was added. The solution was then subjected to dialysis against distilled water using a cellulose membrane with an exclusion limit of 12,400 g mol^−1^, until the conductivity approached that of distilled water. The derivative was then dried through lyophilization.

### 2.4 Determination of the Degree of Oxidation of LBGO

The actual degree of oxidation of the derivatives was determined using the procedure outlined by Zhao and Heindel.^18^ This analysis quantifies the amount of aldehyde groups present in the structure of LBGO10, LBGO30, and LBGO50. The oxidized derivative (100 mg) was dissolved in 25 mL of a 0.25 mol L^−1^ solution of hydroxylamine hydrochloride (NH_2_OH·HCl) and stirred for 24 h. During this period, the aldehyde groups were converted into oximes, and hydrochloric acid (HCl) was released. The amount of HCl was then titrated with a 0.1 mol L^−1^ solution of NaOH, and the change in pH was monitored using a pH meter. All determinations were carried out in triplicate. Each mole of the oxidized monosaccharide unit has two mol of aldehydes, which upon reaction with hydroxylamine hydrochloride, release two mol of HCl. The actual degree of oxidation was calculated by using eq 1:
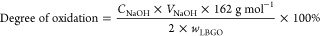
1where *C*_NaOH_ and *V*_NaOH_ are the concentration (mol L^−1^) and volume (*L*) of sodium hydroxide, respectively, *w*_LBGO_ is the weight (g) of the oxidized derivative, and 162 g mol^−1^ is the molar mass of the monosaccharide unit of galactose or mannose.

### 2.5 Preparation of NCEC/LBGO Scaffolds

Aqueous solutions of NCEC, LBGO10, LBGO30, and LBGO50, with a concentration of 2% (w v^−1^), were prepared under magnetic stirring at 50 °C for 24 h. Then, 1 mL of the NCEC solution and 1 mL of one of the oxidized derivatives (LBGO) were pipetted to form hydrogels with a 1:1 volumetric ratio, resulting in three different scaffolds: NCEC/LBGO10, NCEC/LBGO30, and NCEC/LBGO50. The scaffolds were prepared and stored in cylindrical containers, 2.5 cm in diameter and 3.5 cm in height, maintained at room temperature for 24 h for complete cross-linking and then dried by lyophilization.

### 2.6 Scaffold Characterization

#### 2.6.1 Fourier Transform Infrared (FTIR) Spectroscopy

Fourier transform infrared (FTIR) spectra were obtained for locust bean gum and its oxidized derivatives, chitosan, N-carboxyethyl chitosan, and NCEC/LBGO scaffolds in the range of 400−4000 cm^−1^ using a Shimadzu IRTracer-100 spectrometer (Kyoto, Japan). The samples were prepared as pellets by mixing crushed samples with potassium bromide (KBr).

#### 2.6.2 Proton Nuclear Magnetic Resonance (^1^H NMR)

Nuclear Magnetic Resonance (NMR) ^1^H spectra of LBG and its oxidized derivatives were obtained by using a Bruker spectrometer (Avance DRX 500, Karlsruhe, Germany) at 70 °C in deuterated water (D_2_O). 3-(Trimethylsilyl)-1-propanesulfonic acid sodium salt (DSS), at a concentration of 1%, was used as the internal standard for chemical shift.

#### 2.6.3 Gel Permeation Chromatography (GPC)

The peak molar mass (*M*_pk_) of LBG and its oxidized derivatives was determined using gel permeation chromatography (GPC) with a Shimadzu LC-20AD Chromatograph and a RID-10A refractive index detector (Kyoto, Japan). The samples were dissolved in water at a concentration of 1.0 mg mL^−1^, filtered, and injected into a Polysep column (7.8 mm × 300 mm) at a flow rate of 1.0 mL min^−1^ with an aqueous solution of sodium nitrate (NaNO_3_) (0.10 mol L^−1^). The peak molar mass was determined by comparison with a pullulan standard (Shodex P-82) with a molar mass ranging from 5.90 × 10^3^ to 7.88 × 10^5^ g mol^−1^.

#### 2.6.4 Thermogravimetric Analysis (TGA)

Thermogravimetric analysis (TGA) was conducted using a SHIMADZU TGA-50 apparatus (Kyoto, Japan) under a synthetic air atmosphere with a flow rate of 50 mL min^−1^. The temperature range analyzed was 30−800 °C, with a heating rate of 10 °C min^−1^. Scaffolds and precursors were analyzed, approximately 10 mg of each sample, in alumina crucibles.

#### 2.6.5 Porosity Measurement

Porosity was determined using the absolute ethanol infiltration method described in the study of Sarker et al.^19^ Ethanol was utilized as a displacement liquid due to its noninterference with the three-dimensional structure of the scaffolds. Scaffold samples’ diameter and height were measured with a digital caliper, followed by immersion in ethanol and centrifugation for 10 min at 1200 rpm to speed up the infiltration process. The excess ethanol was removed with filter paper, and the scaffolds were weighed repeatedly until a constant mass was obtained. The analysis was performed in triplicate, and the porosity was calculated using eq 2:
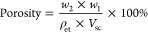
2where *w*_2_ is the weight in grams after immersion in ethanol and *w*_1_ is the weight (g) of the dry scaffold. ρ_et_ is the density of ethanol at 25 °C and *V*_sc_ is the volume (mL) of the scaffold.

#### 2.6.6 Scanning Electron Microscopy (SEM)

The morphology of the scaffolds was visualized by using a Quanta FEG FEI-450 scanning electron microscope (Oregon, USA). The internal porous structure of the scaffolds was exposed by cutting them with a scalpel blade, and then, a thin layer of gold was coated onto their surface.

#### 2.6.7 Swelling Capacity

Swelling capacity tests of the scaffolds were conducted in phosphate buffer, pH 7.4, at room temperature. Scaffolds were weighed and immersed in PBS buffer. They were removed and weighed every 10 min in the first hour and every 30 min thereafter until 3 h had passed. The experiments were performed in triplicate. Swelling capacity was calculated using eq 3:
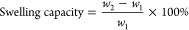
3where *w*_2_ is the weight (g) of the swollen scaffold and *w*_1_ is the weight (g) of the scaffold before swelling.

#### 2.6.8 In Vitro Degradation

The in vitro degradation of the scaffolds was evaluated following the methodology described by Balakrishnan et al.^20^ Briefly, the scaffolds were immersed in 30 mL of phosphate-buffered saline (PBS) at pH 7.4 and kept at 37 °C with constant agitation at 100 rpm in an incubator. Samples were collected at 1, 3, 7, 14, 21, and 28 days after the start of the test. The scaffolds were washed with distilled water to remove salts from the PBS, freeze-dried, and weighed. Triplicate assays were performed to ensure accuracy. The percentage of degradation was calculated using eq 4:
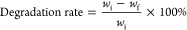
4where *w*_i_ is the initial weight (g) and *w*_f_ is the final weight (g) after the degradation period.

#### 2.6.9 In Vitro Cytotoxicity

The in vitro cytotoxicity of scaffolds and precursor polysaccharides was assessed using the MTT (3-4,5-dimethyl-thiazol-2-yl-2,5-diphenyltetrazolium) assay, which relies on the ability of NADPH-dependent cellular oxidoreductase enzymes to convert MTT, a yellow salt, into formazan, a purple compound,^21^ allowing for the indirect quantification of the percentage of viable cells. Nontumor L929 murine fibroblast mouse cells were cultured in DMEM medium supplemented with 10% fetal bovine serum and 1% antibiotics under an atmosphere containing 5% CO_2_ at 37 °C. Scaffold and precursor polysaccharide extracts were prepared according to ISO 10993-5. Scaffolds sectioned into standardized sizes (1 cm^2^) and 10.0 mg of precursor polysaccharides were sterilized by using UV radiation. DMEM culture medium was used as an extraction vehicle for 24 h at 37 °C under an atmosphere containing 5% CO_2_. L929 cells were seeded in 96-well plates at a concentration of 0.1 × 10^6^ cells mL^−1^. After 24 h of incubation, the culture medium was removed, and 200 μL of scaffold extracts and precursor polysaccharides were added. The plates were then incubated for 69 h at 37 °C and 5% CO_2_. At the end of the incubation period, the supernatant was removed, and 150 μL of a 0.5 mg mL^−1^ MTT solution was added. The plates were further incubated for 3 h, and the precipitate was dissolved with 150 μL of pure DMSO. The absorbance was read at a wavelength of 595 nm using a microplate reader (Molecular Devices, Spectramax 190). The absorbance values were transformed into cell viability from the previously normalized absorbance values based on the mean absorbance of the negative control using the GraphPad Prism version 7.0 program.

### 2.7 In Vivo Wound Healing Study

#### 2.7.1 Animals

Male Swiss mice weighing 25−30 g were used, housed in standard cages under controlled temperature (23 ± 1 °C), a 12 h/12 h light/dark cycle, and had access to water and food ad libitum. All surgical procedures and animal treatments were conducted according to the Guide for Care and Use of Laboratory Animals (National Institutes of Health, Bethesda, MD) and were approved by the Ethics Committee on Animal Use of the Federal University of Ceará (CEUA/UFC number 6081251022—ID 002338).

#### 2.7.2 Experimental Procedure

The animals were anesthetized with 2% xylazine (10 mg kg^−1^, i.p.) and 10% ketamine (90 mg kg^−1^, i.p.). The back of the animals was shaved and cleaned with 5% povidone iodine and 70% alcohol. After antisepsis, an excision of 10 mm^2^ was performed with a surgical punch. The injured area was treated daily with topical application of NCEC/LBGO10, NCEC/LBGO30, NCEC/LBGO50, or saline solution (negative control). On the 14th day, the animals were anesthetized with xylazine (30 mg kg^−1^, i.p.) and ketamine (300 mg kg^−1^, i.p.), and scar tissue was collected for further analysis. Wounds were photographed at day 0, 1, 4, 6, 10, and 14, and the area of the wounds was measured using a digital caliper (Mitutoyo, Resolution: 0.01 mm/.0005”).^22,23^ The percentage of wound contraction was calculated using eq 5:
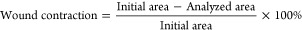
5

#### 2.7.3 Histomorphometric Evaluation

For histopathological evaluation, the tissues were fixed in 10% buffered formalin, sectioned, and embedded in paraffin. Sections (4 μm) were deparaffinized, stained with hematoxylin-eosin, and then examined under a light microscope. The sections were analyzed in a blinded manner. The micrographs of the histological sections were obtained by using a Leica DM2000 microscope (Leica, Wetzlar, Germany) and Leica Application Suite software (Leica, Wetzlar, Germany). Epidermal and dermal thicknesses were measured using a magnification of 400× and 200×, respectively. The analysis was carried out from 12 measurements, with at least 6 slides evaluated per group.^22^

#### 2.7.4 Oxidative Stress

Nitrite/nitrate quantification was performed using the Griess reagent.^24^ Briefly, 100 μL of homogenate (prepared in 1.15% KCl, as reported previously) was mixed with 100 μL of Griess reagent (1% de sulfanilamide and 0.1% naphthyl ethylenediamine dihydrochloride in 1% H_3_PO_4_), which was incubated at room temperature for 10 min. The entire assay was performed on a 96-well plate, and the reading was done at 560 nm. Nitrite/nitrate levels were expressed as μmol L^−1^.

Lipid peroxidation was determined by quantifying the levels of malondialdehyde (MDA) using the thiobarbituric acid reactive substances.^25^ The tissues were homogenized in 1.15% KCl in order to obtain a homogenate at 10%. Then, 100 μL of homogenate was added to 1.5 mL of 1% H_3_PO_4_ and 500 μL of 0.6% thiobarbituric acid. The mixture was then shaken and held at 100 °C for 60 min. The systems were cooled, followed by the addition of 1 mL of n-butanol. The mixture was shaken and centrifuged at 132×*g* for 10 min, and the absorbance was measured at 535 nm. MDA concentration was expressed as nanomoles per milligram of tissue.

The concentration of reduced GSH was determined according to the method described by Sedlak and Lindsay.^26^ Briefly, the tissues were homogenized in 0.02 mol L^−1^ EDTA in order to obtain a homogenate at 10%. Aliquots of 100 μL of the homogenate were mixed with 80 μL of distilled water and 20 μL of 50% trichloroacetic acid (TCA) for protein precipitation. The tubes were centrifuged for 15 min at 3000 rpm at 4 °C. A total of 100 μL of the supernatant was added to 200 μL of 0.4 mol L^−1^ TRIS buffer (pH 8.9) and 10 μL of 0.01 mol L^−1^ 5,5-dithiobis(2-nitrobenzoic acid) (DTNB, Sigma-Aldrich, St. Louis, Missouri, USA). The absorbance was read at 405 nm, and GSH concentration was expressed in μg mg^−1^ of tissue.

#### 2.7.5 Statistical Analysis

Quantitative results were expressed as mean ± standard error of mean (SEM). Data normality was assessed using the Kolmogorov−Smirnov test. Results with parametric distribution were analyzed by one-way Analysis of Variance (one-way ANOVA) followed by Tukey’s post-test, while data obtained from nonparametric distribution were analyzed using Kruskal−Wallis test followed by Dunn’s test. *p* < 0.05 was considered statistically significant. The statistical analysis was performed using GraphPad Prism version 6.0 software (GraphPad Software Inc., La Jolla, CA, USA).

## 3 Results and Discussion

### 3.1 Characterization of Chitosan and NCEC

**Figure 1:**
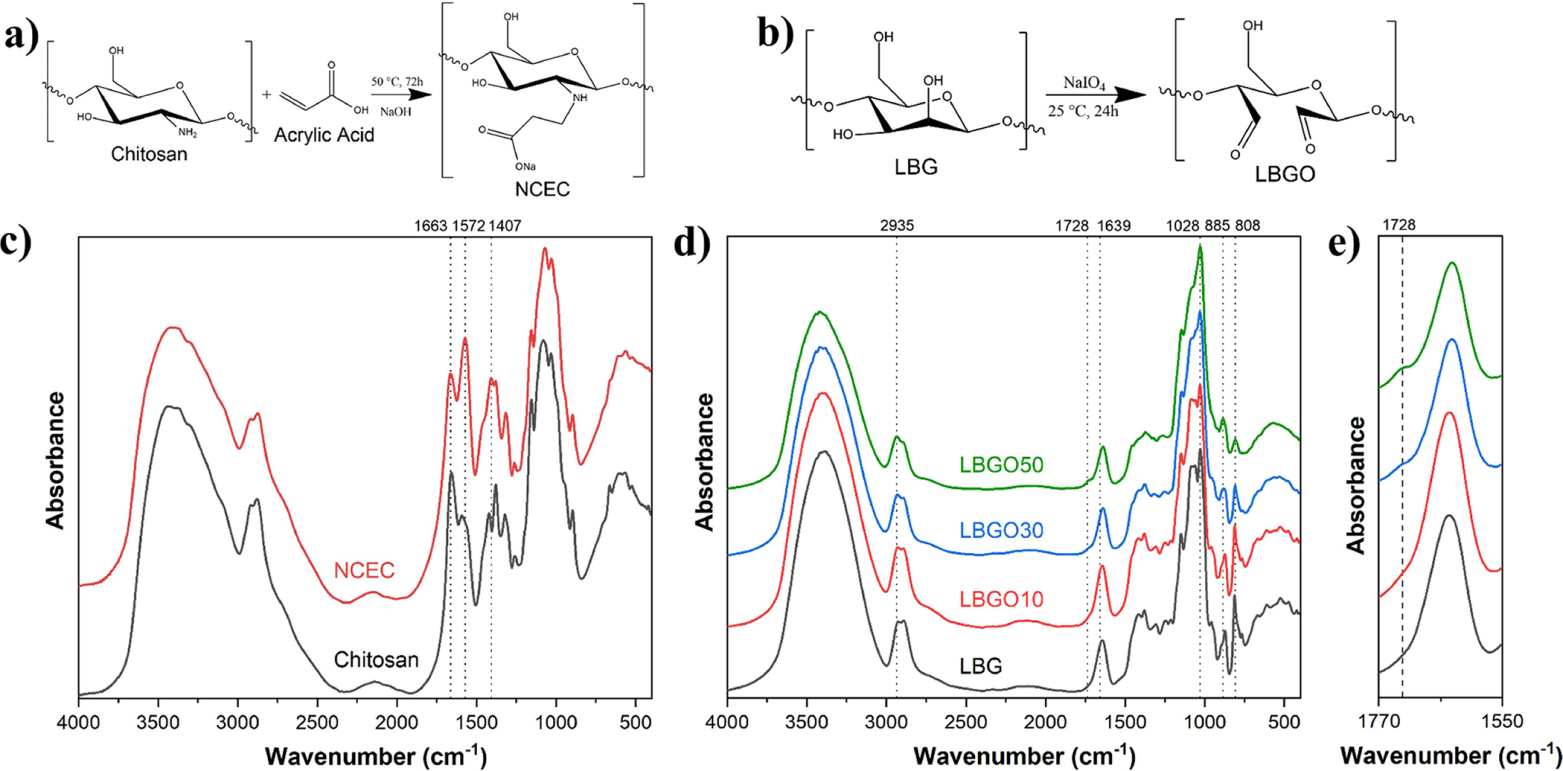
(a) NCEC synthesis reaction; (b) FTIR spectra obtained for chitosan and NCEC; (c) oxidation reaction of locust bean gum with sodium periodate; (d) FTIR spectra of locust bean gum (LBG) and its oxidized derivatives (LBGO10, LBGO30 and LBGO50) and (e) FTIR expanded area (1770−1550 cm^−1^).

The NCEC synthesis (Figure 1a) involves a Michael addition reaction, in which the amino group of chitosan acts as a nucleophilic Michael donor, attacking the β-carbon of acrylic acid, which acts as an electrophilic Michael acceptor. This reaction forms a protonated Michael adduct and yields the carboxylate group (−COO^−^) upon adding NaOH. FTIR analysis (Figure 1b) was conducted to confirm the synthesis of the soluble derivative. The chitosan spectrum displayed a band at 1663 cm^−1^, indicating stretching vibrations related to the presence of residual N-acetyl groups. For the NCEC spectrum, two new peaks appeared at 1572 and 1407 cm^−1^, which were assigned to the asymmetrical and symmetrical stretches of the carboxylate group, respectively.^27^ The observed bands provide evidence of the successful insertion of the carboxylate group via reaction with acrylic acid.

### 3.2 Characterization of LBG and Oxidized Derivatives

The oxidation of locust bean gum by sodium periodate is conducted in an aqueous medium, without the requirement of catalysts, and produces a purified product with only a dialysis step. The reaction proceeds via the attack of periodate ion on vicinal diols, which causes the cleavage of C−C bonds and the formation of aldehyde groups (Figure 1c). The experimental degree of oxidation was determined by potentiometric titration of the HCl released during the reaction between hydroxylamine hydrochloride and the oxidized derivative; the results are presented in Table 1. It can be observed that the experimental values are in agreement with the theoretical values. The oxidation reaction leads to the cleavage of the main chain of the derivatives by breaking the glycosidic bonds. To estimate the molar mass peak (*M*_pk_), gel permeation chromatography (GPC) was used, and a value of 2.67 × 10^6^ g mol^−1^ was obtained for purified locust bean gum. An increase in the degree of oxidation resulted in greater cleavage and lower *M*_pk_, as shown in Table 1.

**Table 1:** Locust Bean Gum Oxidized Derivatives Data

oxidized derivative	theoretical DO (%)^a^	experimental DO (%)^b^	M_pk_ (g mol^−1^)^c^	oxidation yield (%)
LBGO10	10	10.3 ± 0.3	3.65 × 10^5^	68 ± 8.8
LBGO30	30	28.7 ± 0.8	6.02 × 10^4^	68 ± 3.8
LBGO50	50	47.6 ± 2.0	2.35 × 10^4^	67 ± 3.0

a Theoretical degree of oxidation was expressed as a percentage in moles of NaIO_4_ added per total moles of monosaccharide units.

b Experimental degree of oxidation obtained by potentiometric titration.

c Peak molar mass determined by gel permeation chromatography (GPC).

FTIR spectra of locust bean gum and its oxidized derivatives (Figure 1d) were obtained to confirm the occurrence of the oxidation reaction. The FTIR spectra of the oxidized derivatives exhibit the same bands observed in LBG, with the addition of a weak band at 1728 cm^−1^ (Figure 1e), which is assigned to the stretching of the C=O bond of the aldehyde groups. The appearance of this band confirms the presence of oxidized units. Carbonyl typically exhibits strong bands in the infrared region. However, the weak intensity observed in the spectra of the oxidized derivatives can be attributed to the formation of intramolecular and intermolecular hemiacetals, and hydrated forms of the aldehyde group. Notably, the relative intensities of the bands at 2935 and 885 cm^−1^ correspond to the C−H stretching of the aldehyde and hemiacetal structures, respectively, and increase with the degree of oxidation.

**Figure 2:**
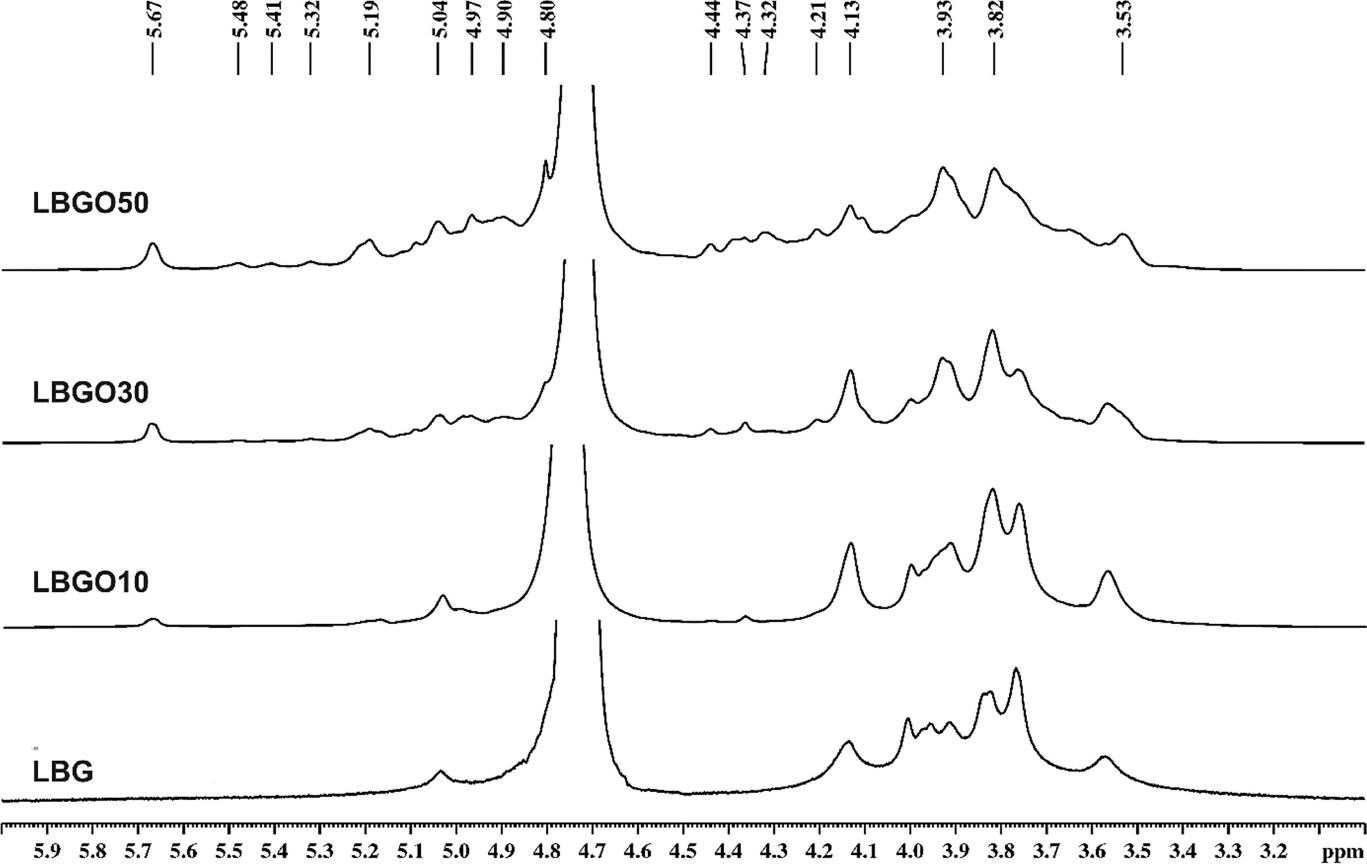
^1^H NMR spectra of LBG and its oxidized derivatives.

^1^H NMR spectra of LBG and the oxidized derivatives (Figure 2) were also obtained to confirm the modification. In the LBGO spectra, new peaks were observed, such as those at 5.67, 5.19, 4.32 ppm, and several others between 5.7 and 4.1 ppm. These peaks may be associated with the hemiacetal structures formed between hydroxyl and aldehyde groups, which can be either intramolecular or intermolecular.^28^

### 3.3 Characterization of Scaffolds

#### 3.3.1 Fourier Transform Infrared Spectroscopy (FTIR)

**Figure 3:**
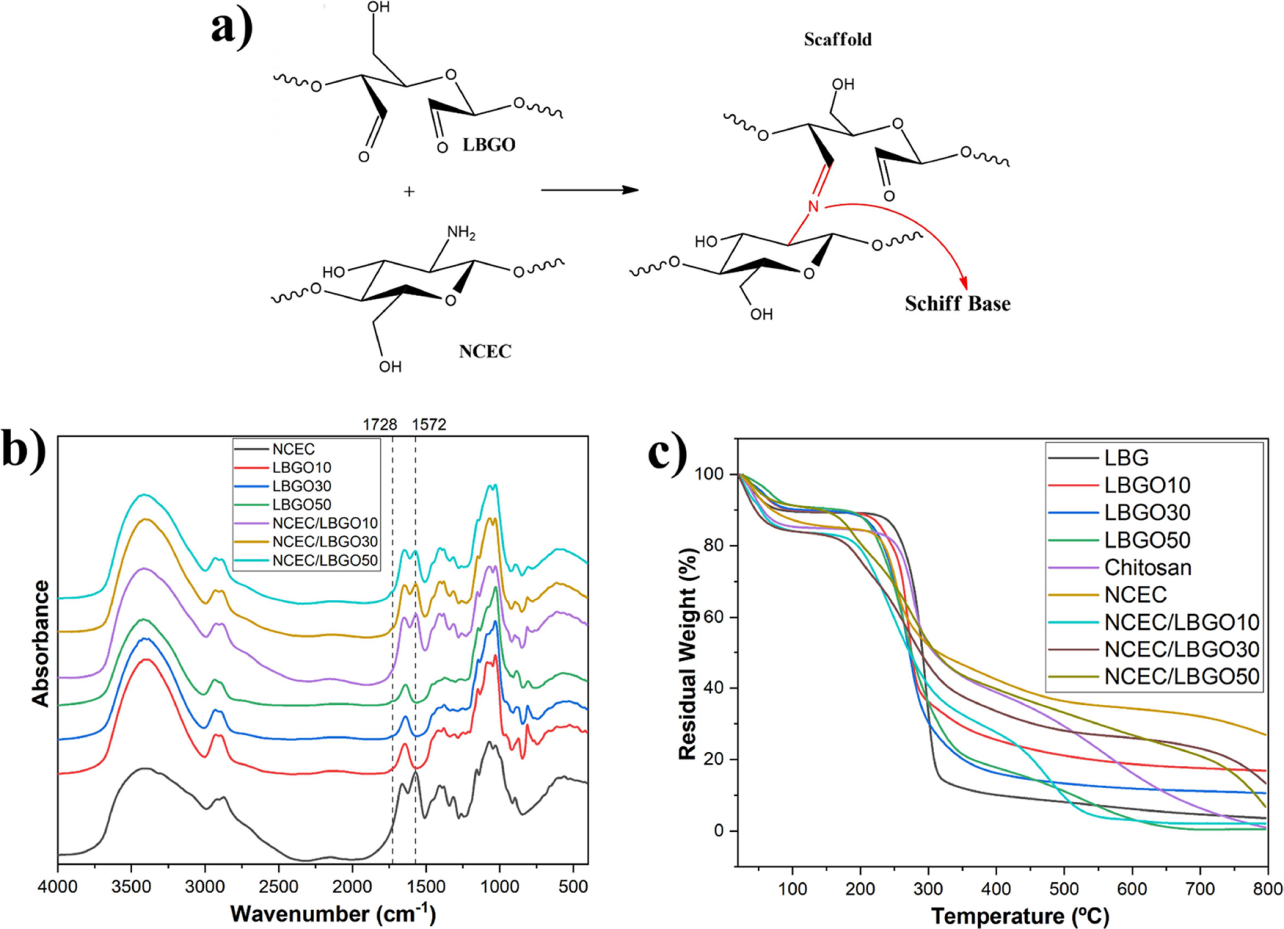
(a) Reaction between NCEC amino groups and LBGO aldehyde groups via the Schiff base reaction, resulting in cross-linking and scaffold formation; (b) FTIR spectra of NCEC, LBGO, and scaffolds; and (c) TGA curves of LBG, oxidized locust bean gum, chitosan, NCEC and scaffolds.

The scaffold formation process involves the cross-linking of aldehyde groups obtained from the oxidation of locust bean gum and the free amino groups of N-carboxyethyl chitosan (Figure 3a). This reaction leads to the formation of a Schiff base (imine, RR′C=NR″) between the polysaccharide chains. Figure 3b displays the spectra of the NCEC, oxidized derivatives, and scaffolds. The formation of the imine bond (C=N) resulting from the Schiff base reaction is indicated by a characteristic band at 1637 cm^−1^, although it was not clearly observed due to overlap with the amide band at 1646 cm^−1^. However, a decrease in the relative intensity of the band at 1728 cm^−1^ attributed to the stretch of the C=O bond of the oxidized locust bean gum, the band related to hemiacetals at 885 cm^−1^, and the band at 1572 cm^−1^ of the N−H stretch of NCEC indicates the occurrence of cross-linking.

#### 3.3.2 Thermogravimetric Analysis

The thermogravimetric analysis (Figure 3c) demonstrated the thermal stability of chitosan, NCEC, locust bean gum, oxidized derivatives, and scaffolds. The results showed a typical profile with a first stage of water loss, followed by one or two degradation stages. These analyses ensure the samples remain stable under sterilization temperatures of up to 100 °C. The first thermal event observed in all samples (polysaccharides and scaffolds) indicates only loss of moisture adsorbed in the polymer chains, which occurs in the range of 25−100 °C. This event occurred with a mass loss that varied from 7 to 17%.

The second thermal event, which consisted of the highest percentage of mass lost in all samples studied (40−72%), occurred around 270−300 °C and refers to the cleavage of the pyranoside ring of polysaccharides, which results in the release of CO_2_ and H_2_O.^29^ It is worth noting that for scaffolds this thermal event is superimposed on a third event, which probably consists of the breaking of the cross-links between NCEC and LBGO resulting from the formation of the Schiff base, which justifies the greater rigidity of the polymer matrix. This hypothesis corroborates data reported in the literature for hydrogels based on chitosan quaternary ammonium salt and oxidized sodium alginate.^30^

In parallel, the characterization of the thermal properties of the NCEC/LBGO10, NCEC/LBGO30, and NCEC/LBGO50 scaffolds showed that the increase in the degree of oxidation of LBGO corroborated the increase in the thermal stability of the biomaterials, which was confirmed by the fact that the degradative events in NCEC/LBGO50 occurred at temperatures higher than those observed in NCEC/LBGO10. This data can be justified by the greater degree of cross-linking of the polymer matrix, which requires more energy for thermal events to occur. Similar results were observed for arabinogalactan-chitosan scaffolds for wound healing and tissue regeneration.^31^ The last thermal event occurred progressively and consisted of the degradation of the remaining organic matter until obtaining a residue at 800 °C, whose percentage value varied from 30 to 5%.

#### 3.3.3 Porosity and Morphology

**Figure 4:**
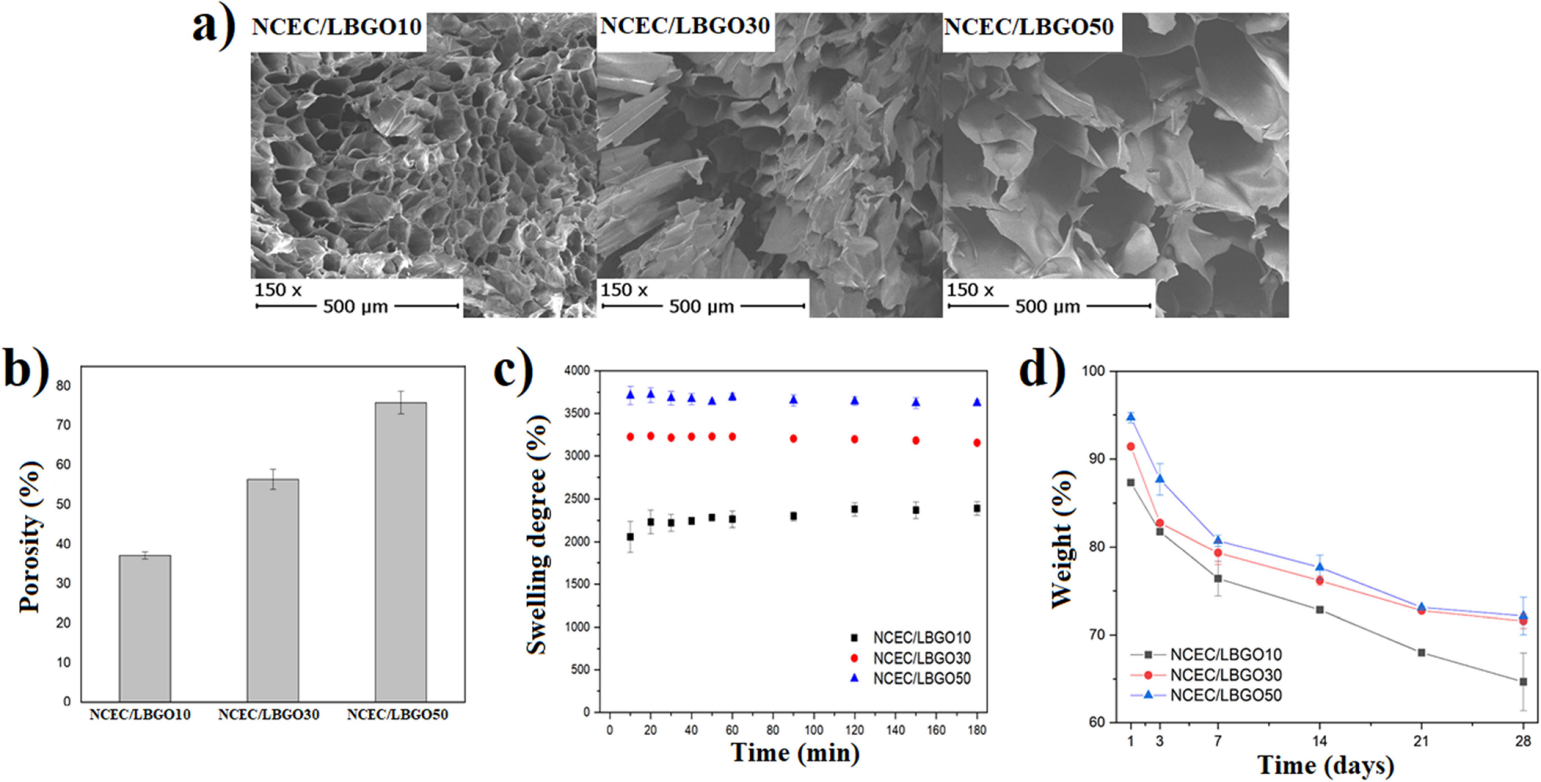
(a) SEM showing the porous structure of the scaffolds; (b) porosity value of the three different scaffolds; (c) swelling degree of scaffold samples in PBS during 180 min; and (d) in vitro degradation of scaffolds in PBS during 28 days.

The porosity of scaffolds is a crucial characteristic for their application in tissue engineering, as it determines the capacity for cell infiltration, tissue formation, and transportation of nutrients and waste products.^32^Figure 4b shows the values obtained for porosity by ethanol infiltration. It is possible to observe that the scaffolds presented a higher porosity value with a higher degree of oxidation: 37.1 ± 0.9% for NCEC/LBGO10, 56.4 ± 2.6% for NCEC/LBGO30, and 75.8 ± 2.9% for NCEC/LBGO50.

Pore size, similar to total porosity, plays a critical role in determining the wound healing potential of scaffolds, enabling tissue growth, vascularization, and facilitating nutrient and gas exchange.^33^ The morphology of the scaffolds was analyzed by using scanning electron microscopy (SEM), and the resulting images are shown in Figure 4a. The scaffolds exhibited a random porous structure, with pore size increasing as the degree of oxidation of the oxidized derivative increased. NCEC/LBGO10 had the smallest average pore size (73 ± 25 μm), which increased to 125 ± 15 μm in NCEC/LBGO30 and 268 ± 66 μm in NCEC/LBGO50. In the literature, it has been reported that there is no significant difference in cell infiltration in scaffolds with pore sizes ranging from 50 to 200 μm.^17^

The formation of the Schiff base resulted in a three-dimensional network, where an increased degree of cross-linking led to a greater number of empty spaces and an increased distance between the NCEC and LBGO polymer chains, which are covalently linked. This structural change contributed to a larger pore diameter and enhanced the porosity. The increase in the degree of cross-linking enhances the mechanical properties of the hydrogels, as evidenced by the storage modulus (*G′*) values, which are 33 Pa for NCEC/LBGO10, 73 Pa for NCEC/LBGO30, and 175 Pa for NCEC/LBGO50 after 10 min of cross-linking (37 °C, 1 Hz, 5 Pa) (Figure S1). This increase is directly related to the higher oxidation degree of LBG, which leads to a greater number of cross-linking points along the polymer chains. The increased availability of aldehyde groups from LBGO facilitates interactions with amino groups from NCEC, thereby reinforcing the hydrogel network and improving its mechanical properties. In addition to the cross-linking degree, the literature highlights that factors such as the biomaterial preparation method, the use of organic solvents, and the nature of the polymer play key roles in modulating this property.^34^

#### 3.3.4 Swelling Capacity

Swelling is another crucial parameter for scaffolds, as it determines their ability to absorb water, which is directly related to the absorption of body fluids. This enables the transport of nutrients and cell proliferation. Furthermore, in the context of wound healing, porous materials with high swelling and absorptive capacity are capable of draining the exudate formed during the inflammation stage, especially in cases of wounds whose infectious component plays a key role in the prognosis of the injury.^35^

Analysis indicates a high swelling capacity as the scaffolds can absorb a mass of water several times greater than their original weight (Figure 4c). The experiment was conducted in phosphate-buffered saline (PBS) since it has a pH and salinity comparable to that of blood and other bodily fluids. The swelling values after 3 h ranged from 2391 ± 80 to 3624 ± 31%, and increased with the degree of oxidation of LBGO used. These results are in agreement with the total porosity and pore size obtained. Comparable values were found in scaffolds prepared with chitosan.^36^ The swelling values remained relatively constant over the 3 h study period, with the scaffolds maintaining their shape.

#### 3.3.5 In Vitro Degradation

The degradation rate of scaffolds for wound healing should align with the regeneration rate of the target tissue. To simulate conditions found in the human body, in vitro degradation of the scaffolds (Figure 4d) was carried out in phosphate-buffered saline (PBS) at 37 °C and 100 rpm, as these parameters replicate physiological conditions. It is observed that the weight loss of the scaffolds increased with decreasing degree of oxidation of the locust bean gum, due to a lower degree of cross-linking. This trend was observed during the 28-day study. The NCEC/LBGO10 hydrogel exhibited the highest weight loss after 28 days (35.3%), while the NCEC/LBGO30 and NCEC/LBGO50 scaffolds lost 28.4 and 27.8% of their weight, respectively. Despite weight loss, the scaffolds retained their three-dimensional structure after the degradation period.

The increase in LBGO oxidation corroborates the greater cross-linking of the polymeric matrix via the formation of Schiff bases; this process increases the hardness of the polymer matrix and may explain the greater swelling capacity and resistance to degradation of NCEC/LBGO50 when compared to other scaffolds. The in vitro degradation profile of NCEC/LBGO-based scaffolds was comparable to values reported in the literature for wound healing scaffolds. For instance, methacryloyl carboxymethyl chitosan exhibited a degradation of 25% over 15 days, while polypyrrole/chitosan/collagen/poly(ethylene oxide) showed degradation ranging from 26 to 28% over 21 days.^37,38^

These results indicated that NCEC/LBGO-based scaffolds have potential for use as wound dressings, as they would be able to remain in the wound bed during all stages (hemostasis, inflammation, proliferation, and remodeling) of the acute wound healing process, which takes around 14−21 days.^22^ Although the stability and long-term storage study of NCEC/LBGO scaffolds was not carried out, after the polymeric dispersion was subjected to the freeze-drying process, the biomaterials obtained presented high physical resistance and can be stored under laboratory conditions (humidity: 40 ± 2%; temperature: 25 ± 3 °C) for several months. Additionally, the in vitro degradation study showed that even in humid conditions, these formulations resist degradative processes, especially at higher degrees of LBGO oxidation.

#### 3.3.6 Wound Dressing Applications

There are several reports on the synthesis, characterization, and use of chitosan-based biomaterials for wound dressing applications, especially formulations obtained from the formation of Schiff bases or physical cross-linking.^39−41^ Thus, Table 2 presents a comparison between the different physicochemical properties of these biomaterials reported in the literature and those observed in this work for NCEC/LGBO scaffolds. In general, it is observed that the data obtained for NCEC/LBGO are similar or more advantageous than those reported, which makes them promising strategies for conducting trials in the preclinical context.

**Table 2:** Comparison between the Physicochemical Properties Reported in the Literature for Other Biomaterials and Those Observed in This Work for N-Carboxyethyl Chitosan/Oxidized Locust Bean Gum Scaffolds

			property studied	
biomaterial	composition	reference	reported in literature	present work
hydrogel	gallic acid-grafted soybean protein isolate + oxidized dextran	(37)	swelling capacity: 40−120%	swelling capacity: 2000−3600%
hydrogel	chitosan quaternary ammonium salt + oxidized sodium alginate + glycerol	(30)	swelling capacity: 500−3500%	
hydrogel	oxidized starch + chitosan	(38)	swelling capacity: 100−400%	
hydrogel	oxidized dextran + chitosan	(20)	swelling capacity: 80−90%	
scaffold	chitosan + gelatin + nanohydroxyapatite	(32)	swelling capacity: 10−30%	
hydrogel	oxidized pectin + gelatin	(37)	swelling capacity: 634−2655%	
hydrogel	oxidized sodium alginate	(40)	swelling capacity: 80−130%	
scaffold	oxidized cashew gum + carboxyethyl chitosan	(15)	porosity: 25−65%	porosity: 37−75%
scaffold	arabinogalactan + chitosan	(31)	porosity: 66−87%	
cryogel	oxidized dextran + chitosan + polydopamine nanoparticles + human-like collagen	(34)	porosity: > 90%	
hydrogel	oxidized chondroitin sulfate + gelatin	(17)	pore size: 50−200 μm	pore size: 73−268
scaffold	oxidized cashew gum + carboxyethyl chitosan	(15)	pore size: 143−213 μm	
scaffold	chitosan + eggshell + vitamin D	(30)	pore size: 50−200 μm	
scaffold	chitosan + gelatin + nanohydroxyapatite	(32)	pore size: 150−300 μm	
cryogel	oxidized dextran + chitosan + polydopamine nanoparticles + human-like collagen	(34)	pore size: 96−122 μm	
hydrogel	arabic gum + pectin + naringin	(43)	pore size: 80−281 μm	
hydrogel	oxidized dextran + chitosan	(20)	weight remaining in degradation study: 43% (6 weeks)	weight remaining in degradation study: 27−35% (28 days)
scaffold	chitosan + gelatin + nanohydroxyapatite	(32)	weight remaining in degradation study: 50−5% (14 days)	
hydrogel	polylysine + dextran	(38)	weight remaining in degradation study: 20% (9−20h)	

Hydrogels, scaffolds, cryogels, and chitosan-based films for wound treatment stand out in the literature for this purpose. Chitosan or its derivatives are associated with other biopolymers, bioactives, or nanoparticles to modulate their physicochemical and pharmacological properties. Based on the data presented in Table 2, it is observed that the choice of the type of material, as well as the components associated with chitosan, are key factors that directly influence its potential for application as a biomaterial. Regarding physicochemical properties, swelling capacity, morphology, and in vitro degradation profile are profoundly affected by these parameters. As for pharmacological activities, in addition to the wound healing potential, antimicrobial and antioxidant activities are the most investigated. Based on data from the characterization of physicochemical properties, in vitro and in vivo assays were conducted with NCEC/LBGO10, NCEC/LBGO30, and NCEC/LBGO50 scaffolds.

### 3.4 Biological Characterization

#### 3.4.1 In Vitro Cytotoxicity

**Figure 5:**
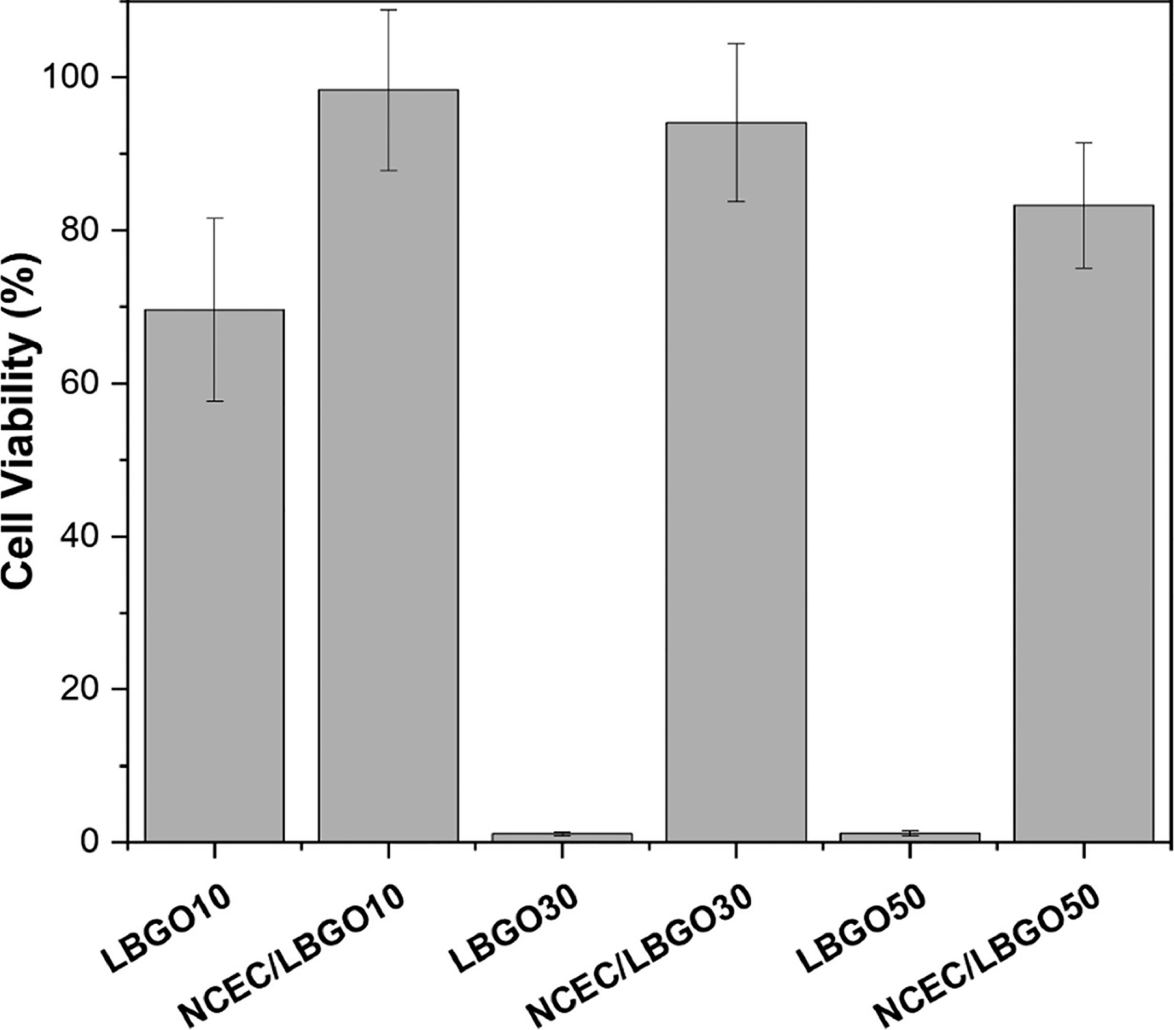
Cell viability of L929 fibroblast cells after 72 h of incubation with scaffolds and precursor polysaccharides using the MTT assay.

Figure 5 demonstrates the results of the in vitro cytotoxicity of oxidized LBG and the scaffolds against L929 murine fibroblasts. LBGO10 exhibited a cell viability of 69.6%, while LBGO30 and LBGO50 showed a significant decrease to only 1.1 and 1.2%, respectively. This decrease is attributed to the increase in the concentration of free aldehyde groups, which can form covalent bonds with nucleophilic sites in biological tissues and impair the functions of enzymes, DNA, structural proteins, and other macromolecules, leading to cellular processes inhibition and cytotoxicity.^42^ On the other hand, all hydrogels presented high cell viability, with NCEC/LBGO10 showing the highest with 98.4%, NCEC/LBGO30 showing 94.1, and 83.3% for NCEC/LBGO50. The free NH_2_ groups in N-carboxyethyl chitosan react quickly with the aldehyde groups of LGBO. Chitosan has a degree of deacetylation of 81%, and its modification to NCEC introduces carboxyethyl groups in approximately 12.5% of the amine units. Consequently, about 68.5% of the monosaccharide units in NCEC retain free amines, allowing the formation of Schiff base bonds with the aldehyde groups of LBGO. LBGO exhibits oxidation degrees of 10.3, 28.7, and 47.6% of its monosaccharide units, with each unit containing two aldehyde groups. Thus, NCEC can bind to a maximum of 34.2% of the monosaccharide units in LBGO, leading to the presence of some free aldehyde groups in the NCEC-LBGO50 scaffolds. The Schiff base formation in the cross-linking process binds most of the aldehydes present in the chains of the oxidized derivatives, thus inhibiting cytotoxicity.

#### 3.4.2 In Vivo Wound Healing Potential

**Figure 6:**
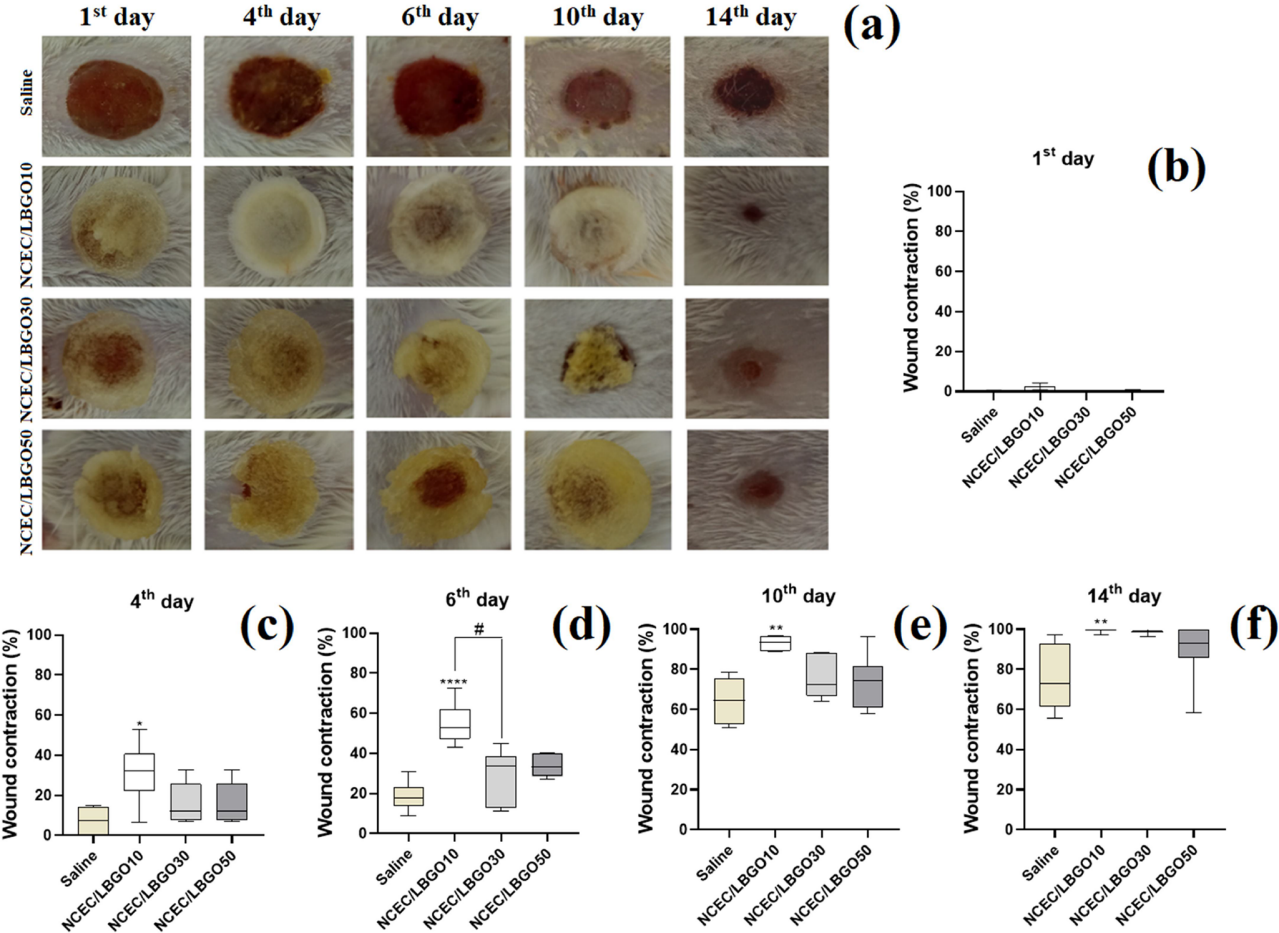
Aspect of skin wounds (a) and evaluation of wound contraction on the (b) 1st, (c) 4th, (d) 6th, (e) 10th, and (f) 14th day of experimental protocol. Results were expressed in box-plot, showing individual data, as well as maximum and minimum values. Where, *p < 0.05 vs Saline, **p < 0.01 vs Saline, ****p < 0.0001 vs Saline and #*p* < 0.05 vs NCEC/LBGO30 according to Kruskal−Wallis followed by Dunn’s test.

The macroscopic evaluation of the wounds showed phlogistic signs of inflammation, such as edema and hyperemia (Figure 6a). On the first day, only the muscle fascia was observed. In contrast, on the fourth day, it was possible to verify crust formation, the presence of exudate, mainly in the Saline group, and the beginning of the re-epithelialization process. From the sixth day, the injuries were less edematous, which made the proliferation phase of the wound healing process clear. On the 10th and 14th days, the lesions showed significant improvement, with slight or absence of edema and characteristic aspect of granulation tissue and new epithelium. On the first day of wound contraction analysis, there was no significant difference between any group (*p* > 0.05) (Figure 6b). On the fourth day, NCEC/LBGO10 (30.94 ± 5.71%) differed significantly (*p* < 0.05) from the saline group (7.17 ± 2.52%) (Figure 6c). On the sixth day, NCEC/LBGO10 (55.04 ± 3.76%) showed more significant wound contraction than the saline (18.45 ± 2.38%) and NCEC/LBGO30 (30.31 ± 1.98%) groups (Figure 6d). On the 10th and 14th days of the experimental protocol, only the NCEC/LBGO10 group (93.27 ± 1.26 and 99.50 ± 0.36%, respectively) differed significantly (*p* < 0.05) from the treatment with Saline group (64.32 ± 4.24 and 75.88 ± 6.01%, respectively) (Figure 6e,f). It is important to mention that some animals in the NCEC/LBGO10 group had completely healed wounds, while in the other groups, the animals showed evidence of injury. These results indicated that topical treatment with the NCEC/LBGO10 formulation can accelerate healing, indicating its potential for wound dressing applications. The porous structure of scaffolds is similar to the extracellular matrix, which contributes to cell migration and supports the tissue under repair. Furthermore, studies point to the ability of these systems to stimulate angiogenesis and gas exchange.^43^

This may explain scaffolds’ better wound healing effect based on NCEC/LBGO association compared to the Saline treatment. The wound healing effect of polysaccharide-based materials has already been extensively reported in the literature.^22,44,45^ However, these macromolecules have disadvantages such as their high hydrophilicity and limited mechanical properties, which can be improved through chemical modifications. It should be mentioned that NCEC/LBGO30 and NCEC/LBGO50 formulations did not dissolve in the wound bed. In contrast, NCEC/LBGO10 forms a hydrogel, which can be explained by the lower cross-linking rate between the LBGO and NCEC chains in this system, which reduces its hardness and increases its hydrophilicity. As noticed in this study, Chetouani et al.^46^ also observed that systems based on gelatin and oxidized pectin with lower cross-linking present better wound healing properties. In addition, the scaffolds prepared in this work showed a higher percentage of wound contraction on the 14th day than those reported for hydrogels based on oxidized dextran and thiolated ε-poly l-lysine.^47^

#### 3.4.3 Histomorphometric Analysis

**Figure 7:**
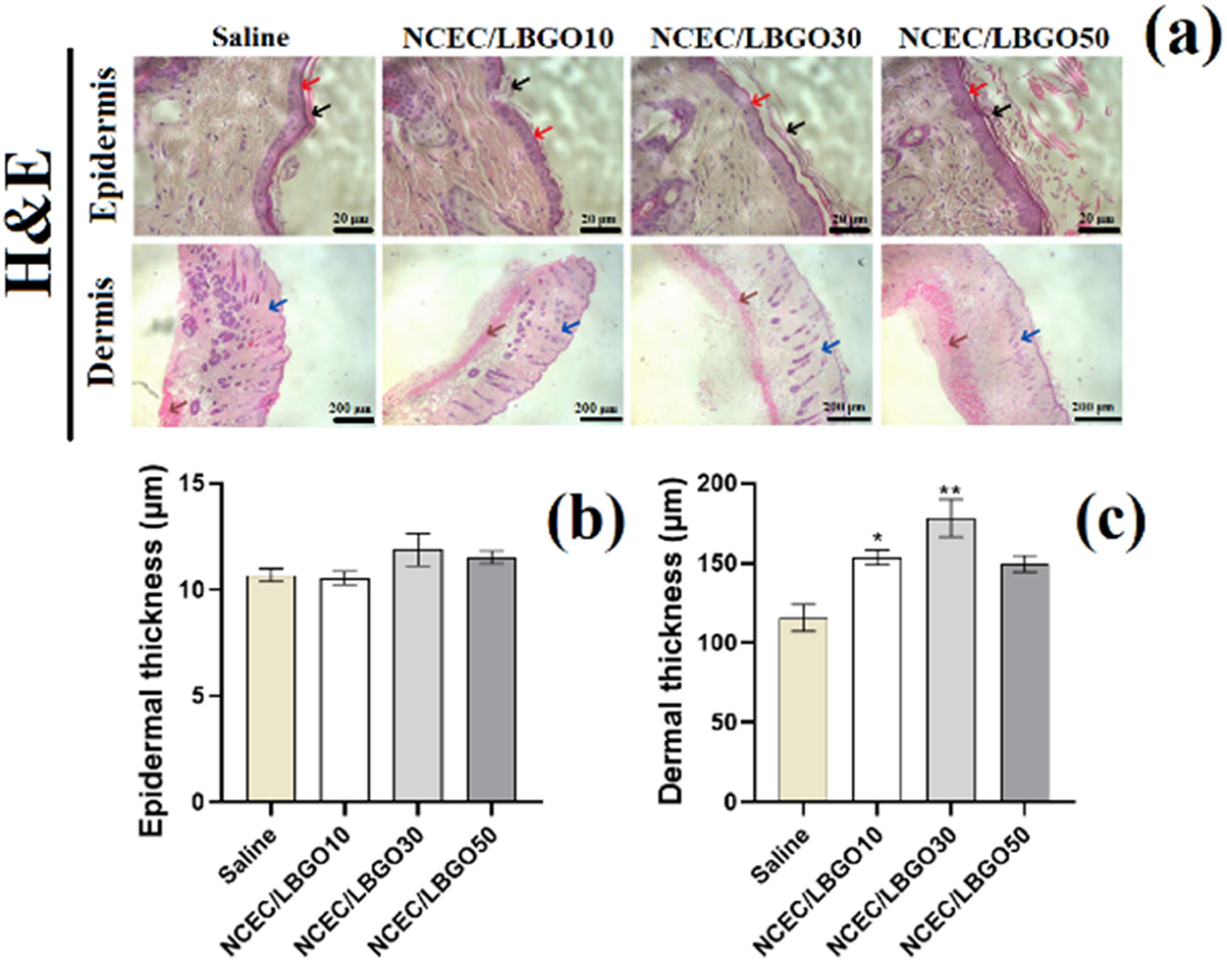
(a) Histological analysis of the regenerated skin tissue after 14 days of treatment and (b) evaluation of epidermal and (c) dermal thickness. Results were shown as mean ± SEM. *p < 0.05 vs saline and **p < 0.01 vs saline according to one-way ANOVA followed by Tukey test as post hoc. Red arrow: epidermal layer; black arrow: keratin; blue arrow: dermal layer; and brown arrow: muscle fiber.

The results of the histomorphometric analysis are shown in Figure 7. In the histological micrographs (Figure 7a), it is possible to observe the three layers of the skin and the presence of different skin appendages. In the epidermal layer (red arrow), the presence of keratin (black arrow) is notable, and its presence is responsible for providing impermeability to water and physical resistance. In the dermis (blue arrow), it is possible to observe many hair follicles, blood vessels, and the differentiation between the papillary and reticular layers. The presence of adipose tissue, characteristic of the hypodermis, and muscle fibers (brown arrow) is also observed.

The results of the histomorphometric evaluation for the epidermis and dermis are shown in Figure 7b,c. No significant difference (*p* > 0.05) was observed in the thickness of the epidermis for the topical treatment with the different experimental groups. On the other hand, dermal thickness was significantly increased (*p* < 0.05) by the administration of NCEC/LBGO10 (153 ± 4.6 μm) and NCEC/LBGO30 (178 ± 11.6 μm) formulations in the wound bed when compared to the Saline group (115 ± 8.4 μm). The effect of scaffolds on dermal thickening may be indicative of greater collagen deposition, since this macromolecule, together with elastin, fills this layer and favors its greater tensile strength and firmness.^48^

#### 3.4.4 Assessment of Oxidative Stress

**Figure 8:**
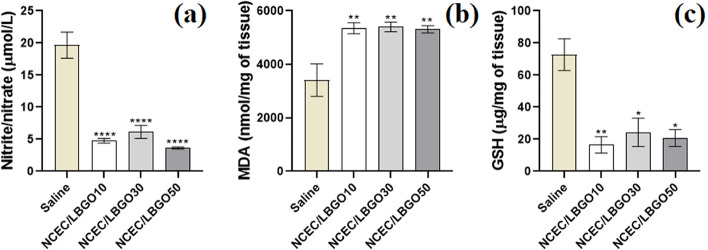
Antioxidant potential of N-carboxyethyl chitosan/oxidized locust bean gum scaffolds: (a) nitrite/nitrate, (b) malondialdehyde (MDA), and (c) reduced glutathione (GSH) levels. Results were shown as mean ± SEM. *p < 0.05 vs saline, **p < 0.01 vs saline, and ****p < 0.0001 vs saline according to one-way ANOVA followed by the Tukey test as post hoc.

The wound healing process progresses with the release of reactive oxygen (ROS) and nitrogen (RNS) species due to the recognition of pathogens or molecular patterns associated with damage by cells residing in the skin tissue (mast cells, dendritic cells, and macrophages) and leukocytes, mainly during the inflammation phase.^45^ The intense release of these mediators hampers the healing process by contributing to tissue injury. At the same time, it also consists of a defense mechanism against infectious agents that may settle in the injured environment and lead to its chronification.^49^Figure 8 presents the results of the assessment of oxidative stress in skin tissue.

Nitric oxide at low concentrations can promote cell proliferation, increased tissue nutrition, and collagen deposition during wound healing. However, at high concentrations, this mediator can react with reactive oxygen species, such as superoxide anion (O^2−^), leading to the formation of peroxynitrite ion (ONOO^−^), a strong endogenous oxidizing agent.^50^ The NCEC/LBGO10 (4.77 ± 0.36 μmol L^−1^), NCEC/LBGO30 (6.15 ± 1.02 μmol L^−1^) and NCEC/LBGO50 (3.66 ± 0.16 μmol L^−1^) formulations significantly reduced (*p* < 0.05) nitrite/nitrate levels when compared to the Saline group (19.67 ± 2.04 μmol L^−1^) (Figure 8a).

MDA is the end product of lipid peroxidation and serves as a laboratory marker for the assessment of this process. High levels of MDA activate pro-inflammatory cytokines, like TNF-α and IL-1β.^51^ Wound treatment with NCEC/LBGO10 (5354 ± 204 nmol mg^−1^ of tissue), NCEC/LBGO30 (5403 ± 179 nmol mg^−1^ of tissue), and NCEC/LBGO50 (5311 ± 135 nmol mg^−1^ of tissue) formulations increased (*p* < 0.05) tissue levels of MDA compared to the Saline group (3409 ± 611 nmol mg^−1^ of tissue) (Figure 8b).

Glutathione is a tripeptide (glutamic acid, glycine, and cysteine). It is one of the main endogenous antioxidant agents due to the presence of thiol groups (−SH) that can react with electrophilic species and prevent the destabilization of macromolecules, such as proteins and nucleic acids.^52^ On the other hand, GSH levels in skin tissue were significantly reduced (*p* < 0.05) by the formulations NCEC/LBGO10 (16.52 ± 5.2 μg mg^−1^ of tissue), NCEC/LBGO30 (24.32 ± 8.8 μg mg^−1^ of tissue) and NCEC/LBGO50 (20.81 ± 5.3 μg mg^−1^ of tissue) when compared to Saline treatment (72.77 ± 9.9 μg mg^−1^ of tissue) (Figure 8c).

Treating wounds with gum arabic/pectin-based hydrogels increased tissue oxidative stress (increased the levels of nitrite/nitrate and MDA and reduced the level of GSH). However, incorporating the flavonoid naringin attenuated this effect.^52^ Similar results were reported for cottons based on carboxymethyl chitosan for wound healing applications.^53^

In addition to presenting better results in the preclinical assay, being able to accelerate wound closure, attenuate tissue oxidative stress, and increase the thickness of the dermis, the NCEC/LBGO10 scaffold presented lower cytotoxicity against L929 fibroblast cells. The physicochemical characterization showed that this biomaterial presented porosity and pore sizes compatible with cell migration and good swelling capacity, and was able to maintain its structure in the in vitro degradation test. These characteristics confront what is often reported as a disadvantage for bioactive dressings based on hydrocolloids, such as low exudate drainage capacity, disruption of the wound bed after removal, cytotoxicity, low mechanical resistance, and low adhesion to the wound.^54^

Based on these results, it is estimated that the NCEC/LBGO10 formulation has greater potential to be used as a wound dressing. Bioactive compounds can be incorporated into this system to improve wound healing activity and assemble other biological activities. preferably with antioxidant activity to oppose ROS production and avoid the depletion of endogenous antioxidant agents.

## 4 Conclusions

The present study reports the characterization of the physicochemical and morphological properties of scaffolds based on N-carboxyethyl chitosan and oxidized locust bean gum. Semisynthetic derivatives of chitosan and locust bean gum were successfully synthesized and led to the formation of biomaterials via Schiff base reaction. The scaffolds showed high swelling capacity, porosity, and resistance to degradation under physiological conditions. Such properties were modulated based on the degree of oxidation of locust bean gum and showed promise considering their use for wound healing due to having pores capable of promoting cell migration and a physical structure compatible with exudate drainage. The MTT assay showed that the scaffolds did not significantly affect the cell viability of L929 fibroblast cells, indicating their low cytotoxic potential.

The evaluation of the healing activity in the experimental excisional wound model in mice showed that the topical use of these biomaterials is capable of accelerating the healing of the injured tissue as well as improving the physical characteristics of the recently formed tissue, with regard to increasing the thickness of the epidermis and dermis layers. Furthermore, the scaffolds were able to reduce the levels of nitrite/nitrate and MDA, two mediators related to increased tissue oxidative stress, indicating their in vivo antioxidant activity. Thus, this research reports the development of biomaterials with potential for wound treatment as well as providing support for carrying out subsequent studies seeking, for example, the entrapment of bioactives in these matrices and their use as a drug release system.

## Supporting Information Available

Evolution of the storage modulus (*G*′) and loss modulus (*G*″) over time for all hydrogels (PDF)
