# Supplementary material for: Effective Dressing: Development of N-Carboxyethyl Chitosan/Oxidized Locust Bean Gum Scaffolds
Source: ACS Omega. 2025 Apr 21;10(16):16717–30. doi: 10.1021/acsomega.5c00521 (PMC12044468; doi:10.1021/acsomega.5c00521)

Supporting Information

Effective Dressing: Development of N-carboxyethyl Chitosan/Oxidized Locust Bean Gum Scaffolds

Luis F. S. Araujo[1]*, Carlos R. do N. Ferreira*[1]*, Gisele S. de Araújo*[2]*, Ana J. Araújo*[2]*, José D. B. Marinho-Filho*[2]*, Ana B. N. Lima*[1]*, André T. de F. F. Dias*[1]*, Matheus da S. Campelo*[1]*, Renata F. de C. Leitão*[3]*, Maria E. N. P. Ribeiro*[1]*, Regina C. M. de Paula*[1]*, Judith P. A. Feitosa*[1]*, Jeanny da S. Maciel*[1]*

[1]Departamento de Química Orgânica e Inorgânica, Universidade Federal do Ceará, Av. Mister Hull s/n, 60455-760, 60455-760, Fortaleza, Brazil

[2]Laboratório de Cultura de Células do Delta, Universidade Federal do Delta do Parnaíba, Av. São Sebastião 2819, 64202-020, Parnaíba, Brazil

[3]Departamento de Morfologia, Faculdade de Medicina, Universidade Federal do Ceará, R. Delmiro de Farias s/n, 60430-170, Fortaleza, Brazil

*Corresponding Author: jeannydsm@ufc.br

Figure S1: Evolution of the storage modulus (G') and loss modulus (G'') over time for all hydrogels at 37 °C, with a frequency of 1 Hz and a shear stress of 5 Pa on an AR 550 rheometer from TA Instruments (Delaware, USA) with the following parameters: cone-plate geometry (40 mm diameter, 1° angle, 28 µm gap).

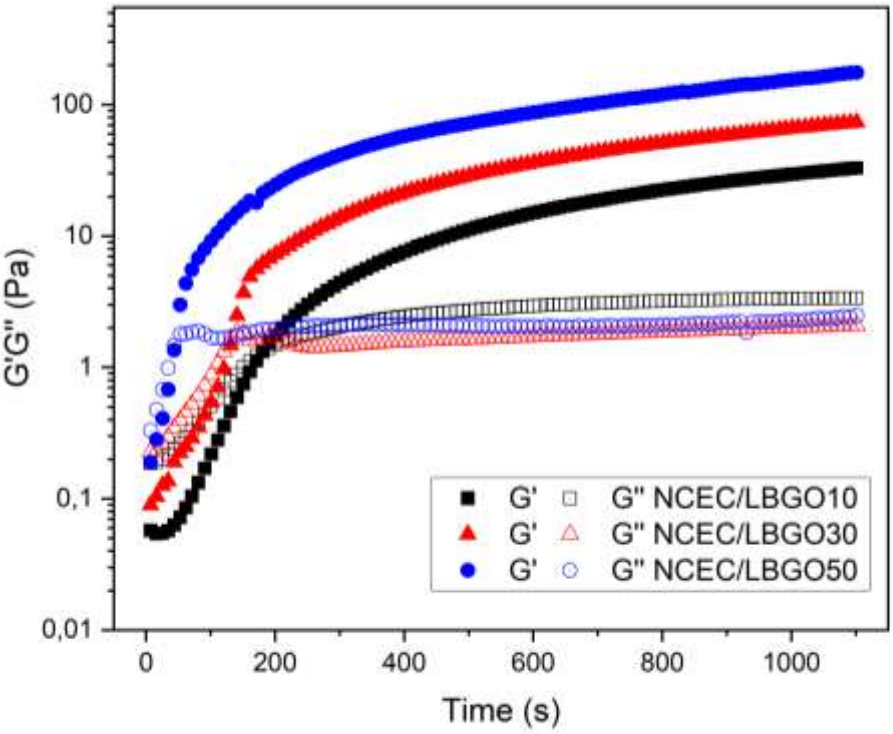

Supplement: Supplementary file 1 — ao5c00521_si_001.pdf [file ao5c00521_si_001.pdf]
